# Supplementary figures and images for: A novel approach towards a histone replacement system in Tetrapods
Source: PLoS One. 2026 Feb 10;21(2):e0342014. doi: 10.1371/journal.pone.0342014 (PMC12890102; doi:10.1371/journal.pone.0342014)

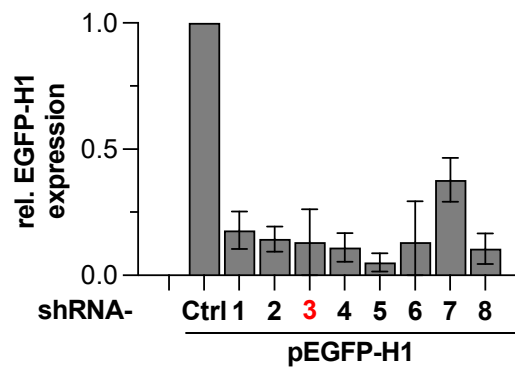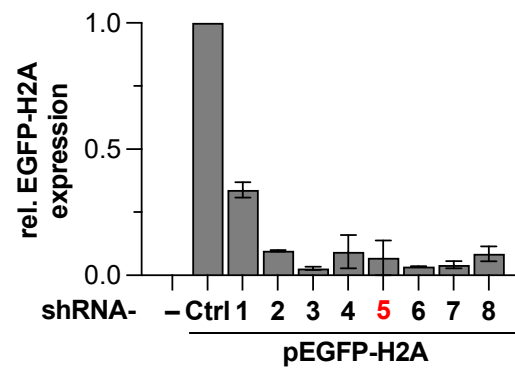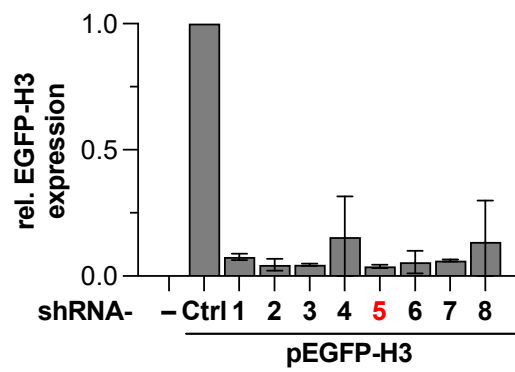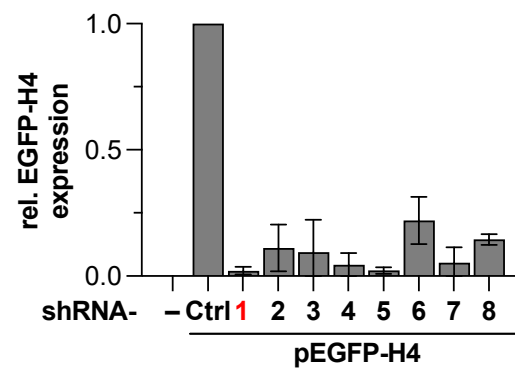

Supplement: S1 Fig — Expression vectors for EGFP fused with the respective canonical chicken histones were generated. The individual fusion proteins were co-expressed in HEK293T cells with plasmids encoding different histone shRNAs or a control shRNA. After 2 days, total RNA was extracted, and mRNA levels were measured by qPCR against EGFP and normalized to the control. Data from 3 independent biological replicates are shown as means ± SD, with all samples normalized to their respective shRNA control. The shRNAs highlighted in red were used for further experiments. (PDF) [file pone.0342014.s001.pdf]
