# Supplementary material for: A novel approach towards a histone replacement system in Tetrapods
Source: PLoS One. 2026 Feb 10;21(2):e0342014. doi: 10.1371/journal.pone.0342014 (PMC12890102; doi:10.1371/journal.pone.0342014)

Raw data for Fig. 4B

Western Blot with ECL
imaged with BioRad ChemiDoc MP

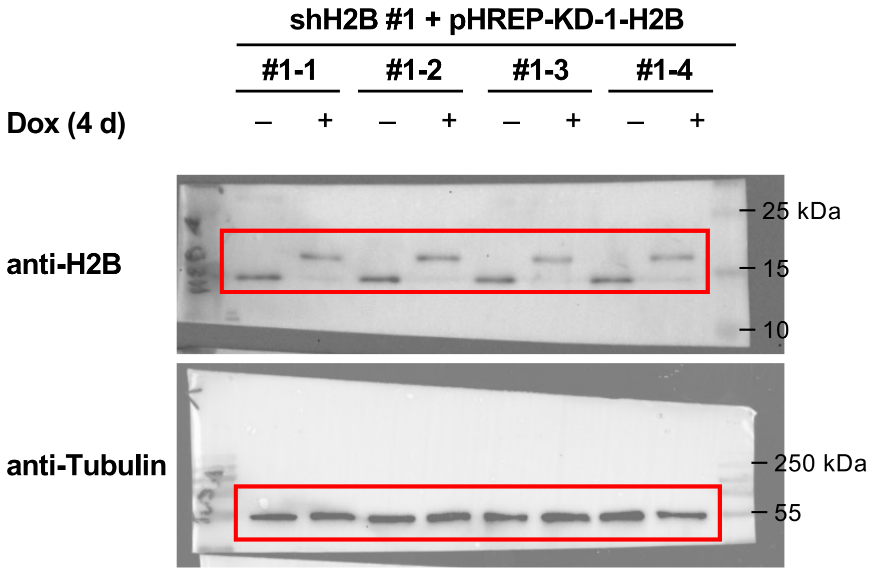

Raw data for Fig. 7C

Western Blot with ECL
imaged with BioRad ChemiDoc MP

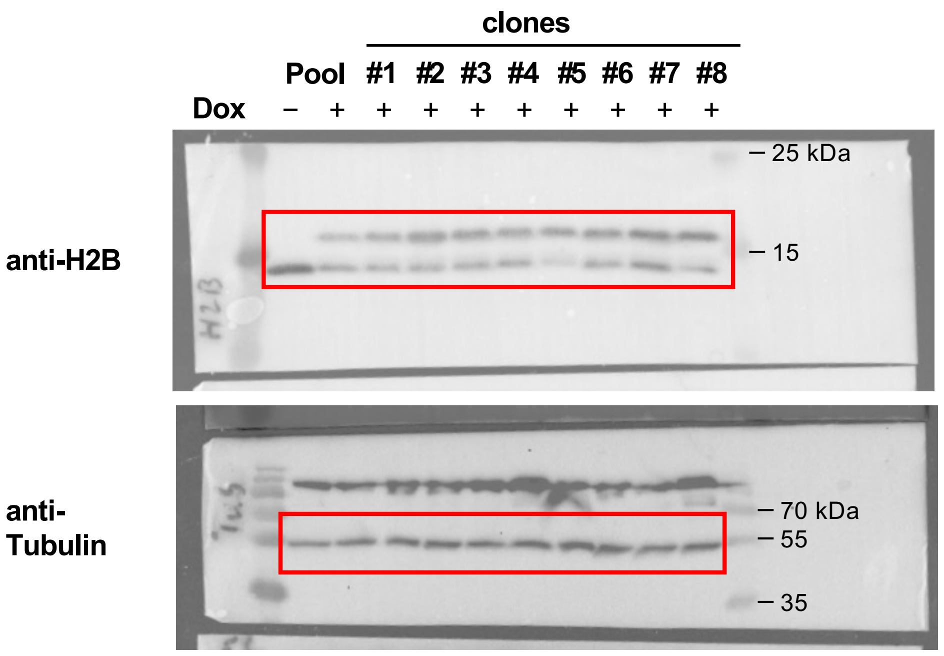

Supplement: S1 File — (PDF) [file pone.0342014.s002.pdf]
