# Supplementary material for: A novel approach towards a histone replacement system in Tetrapods
Source: PLoS One. 2026 Feb 10;21(2):e0342014. doi: 10.1371/journal.pone.0342014 (PMC12890102; doi:10.1371/journal.pone.0342014)
Supplement: Attachment — Submitted filename: Rebuttal letter.docx [file pone.0342014.s005.docx]

| Institute of Biochemistry, Friedrichstrasse 24, 35392 Giessen | Prof. Dr. M. Lienhard Schmitz  Friedrichstrasse 24  35392 Giessen  Tel: +49 641 99-47570/1  Fax: +49 641 99-47589  lienhard.schmitz@biochemie.med.uni-giessen.de  (https://www.uni-giessen.de/en/faculties/f11/departments/biochemistry)  12.01.2026 |
| --- | --- |
| To  Zu Ye, Ph.D.  Academic Editor  PLOS One |  |

Re. Revision of PONE-D-25-37538

Dear Dr. Ye,

Thank you very much for sending us the referees` comments on our manuscript by Pfisterer et al. entitled "A novel approach towards a histone replacement system in Tetrapods". In the meantime we have revised the manuscript according to the suggestions of the reviewers. In the following section, we will provide a point-to-point answer to the questions, criticism and queries raised by the two referees, who really helped to amend this manuscript. Our responses to the reviewers written in blue:

**Reviewer #1:**"I am enthusiastic about this paper..."

Answer: Thank you very much for these positive comments.

**Reviewer #2**:

1) I think that instead of using an inducible promoter to restore the depleted histones that, as authors indicate, does not respect the S-phase natural expression of histones, it would be much more accurate to express them from their natural promoters in a multicopy construct. In this way any potentially perturbing effects of expression out of S-phase would be bypassed at the same time that expression will likely result in the correct amount of protein expressed. Have the authors tried this approach?

Answer: We fully agree with the concern that S-phase-specific expression of the replacement histone would more faithfully recapitulate the endogenous histone expression. We therefore inserted histone stem loops, but this did not improve expression. A second option would be the insertion of Tet operators upstream of the cell cycle-specific histone promoters. Historically, however, the development of the Tet-On system and the optimization of all the different parameters ensuring the tightness and strong inducibility of the system took more than a decade (Paillard et al. Hum Gene Ther. 1998, PMID: 9607408; Colicchia et al. FEBS Open Bio. 2022, PMID: 36062323) and thus lies beyond the scope of this study. We have addressed all these aspects in the revised discussion.

2) In agreement with the authors, I think it is a good approach to introduce landing pads to minimize random integration side effects. However, to make them trustable the landing pads positions in the genome should be mapped and those in the best positions (those having minimal effects) selected.

Answer: We fully agree with this statement. However, since this is merely a proof-of-principle study, we have only determined the copy number of integration sites, but not their genomic locations. A complete characterization would also require to asses whether the integrations are *bona fide* safe harbor sites: genetically stable locations that are not subject to potential (epi)genetic changes and not showing interactions with other genomic loci. In accordance with the reviewer’s suggestions, these aspects have been included in the revised discussion.

3) On one hand, authors indicate that the copy number (but also the variants) of the different histones may be a problem in vertebrates other than chicken. However, when they tested it in human HEK293T cells the approach seemed to work properly, as shown at the mRNA level. It would be very informative if semiquantitative WB analyses for these experiments are included.

Answer: We would like to clarify this point in more detail: In the experiment, the endogenous histones of 293T cells were not downregulated. As described in the manuscript, EGFP-tagged chicken histones were coexpressed with the different shRNAs against the chicken histones in 293T cells to provide a rapid and quantifiable system for the characterization of all the various shRNAs.

Minor points:

On Table 1 there is a mistake. Human histone H1 are 7 somatic isoforms (+4 germline specific isoforms)

Answer: Thank you for spotting this mistake, the table has been corrected accordingly.

In Drosophila the histone replacement systems designed by the group of Robert Duronio (McKay et al, (2015) Dev Cell32, 373-386 and a CRISPR-CAS9 based approach by the group of Guanjun Gao (Zhang, W. et al. (2019) Dev. Cell 48, 406–419) should be also referenced in the introduction.
Answer: The reference from McKay and coworkers was added, the paper by Zhang et al. was already cited.

There are a few typing mistakes.

Answer: We have carefully read through the text again and tried to identify and correct all errors.

**Reviewer #3:**

1) The authors choose to target canonical histones, which is reasonable; however, variant histones are only mentioned in passing and not discussed in the context of the replacement system. The non-canonical histone variants could be an important consideration for many of the histones, as they will likely not be targeted by the shRNAs given sequence differences between the genes. It is possible that the non-canonical histones could compensate for endogenous histones targeted by the shRNA knockdown and complicate interpretation of the resulting data. This would be an important point to highlight in the manuscript.

Answer: We have added these considerations, which apply to all histone replacement systems, to the discussion.

2) The authors demonstrate downregulation of endogenous histone H2B by western blot, which is efficient but incomplete in the first-generation system and relatively inefficient in the second-generation system. The western analyses do not provide information on the genomic localization of remaining endogenous histone and it is possible that the endogenous histone is not uniformly distributed across the genome, but rather collects at higher levels in certain regions compared to others (perhaps even at wild type levels). This could be important for comparative analyses since one could imagine that certain genes may retain endogenous wild type histone rather than a mutant replacement histone, which would have the potential to skew both gene expression and resulting phenotypes. Ideally, the authors would show genome-wide distribution of the endogenous histone in the replacement system; however, this would be challenging since antibodies against H2B would not distinguish between endogenous and exogenous histone. The authors could infer levels of endogenous histone by mapping total histone and exogenous histone based on the epitope tag. At a minimum, this complication should be discussed in the text.

Answer: This point is well taken and overlaps with the comment from Reviewer #3, point 2. Accordingly, we have addressed these important points in the revised manuscript. The revised version considers two distinct levels:

a) At the genomic level, these cell systems will require comprehensive and deep phenotyping, including mapping the integration sites of the landing pads and characterizing the integration regions as safe harbor loci: genetically stable locations that are not subject to potential (epi)genetic changes and do not interact with other genomic loci.

b) At the histone level, it is important to distinguish between endogenously and exogenously expressed histones. In our system, this was achieved through the addition of epitope tags, which allow discrimination between endogenous and re-expressed histones at both the biochemical and genetic levels.

We suggest that such experiments are best suited for follow-up studies building on this proof-of-principle work.

3) The authors use 96-well plates to screen for clones that survive and proliferate following histone replacement. On page 10, the authors note that “most clones showed poor survival.” Is there any concern that the authors are selecting for clones with an abnormal karyotype that bestows a growth advantage? The puro selection is an intentional effort to identify clones that highly express the replacement histone but the puro resistance gene should be effective at very low levels. The persistence of only four clones seems to indicate a harsh selective process from initial histone knockdown. Ensuring that the cells are not abnormal as a result of this process could be important for studies attempting to associate histone modifications with cell physiology.

Answer: We have revised and improved our text, which was indeed somewhat ambiguously phrased, so that this aspect is now more clearly conveyed. It is now described that stable integration of the plasmids for shRNA knockdown is achieved via the neomycin resistance gene, while the addition of puromycin selects for tetracycline-inducible shRNA expression. This approach led to the isolation of four cell clones that ceased to proliferate upon addition of Dox, whereas they grew normally and comparably to wild-type cells in the absence of Dox. The relatively small number of obtained cell clones is likely due to the requirement for very high levels of inducible shRNA expression to downregulate the endogenous histone. As reported in the literature, a *bona fide* proliferation defect occurs only when large amounts of the histone are depleted (Corcoran et al. Plant Cell 2022, PMID: 35879829)

4) As noted above, knockdown of endogenous histone is inefficient in the second generation system. The authors should provide replacement efficiencies as shown in Figure 4C and discuss the implications if replacement is weak.

Answer: As suggested by the reviewer, we quantified the H2B replacement efficiency, which is now shown in the new Fig. 7D. The results are discussed in the revised version of the manuscript.

Minor point
There are different kinds of histone replacement systems (i.e., partial vs complete), which are discussed in PMID: 35853806. This is a minor semantics point, but it seems that the system outlined in this manuscript should be defined as partial to avoid confusion with complete systems.

Answer: This point is correct, and we therefore now state that a partial histone replacement system has been developed.

We thank the reviewers for the time and effort they have invested to further improve the conceptual, technical and didactical parts of the manuscript. All authors have seen the final version of the manuscript and concur with its content We are confident that we have addressed all major issues and hope that the manuscript has experienced strong amendment so that it can be considered further for publication.

With best wishes,

Lienhard Schmitz & Maximilian Pfisterer
